# Supplementary material for: Filopodia rotate and coil by actively generating twist in their actin shaft
Source: Nat Commun. 2022 Mar 28;13:1636. doi: 10.1038/s41467-022-28961-x (PMC8960877; doi:10.1038/s41467-022-28961-x)
Supplement: Supplementary file 1 — Supplementary Information [file 41467_2022_28961_MOESM1_ESM.pdf]

Filopodia rotate and coil by actively generating twist in their actin shaft

Natascha Leijnse**,† Younes Farhangi Barooji**,† Mohammad Reza Arastoo,† Stine Lauritzen Sønder,‡ Bram Verhagen,† Lena Wullkopf,¶ Janine Terra Erler,¶ Szabolcs Semsey,† Jesper Nylandsted,§ Lene Broeng Oddershede,† Amin Doostmohammadi,*,† and Poul Martin Bendix*,†

†*Niels Bohr Institute, University of Copenhagen, 2100 Copenhagen, Denmark*

‡*Membrane Integrity, Danish Cancer Society Research Center, Strandboulevarden 49, 2100 Copenhagen, Denmark*

¶*Biotech Research and Innovation Centre (BRIC), University of Copenhagen, Ole Maaløes Vej 5, 2200 Copenhagen, Denmark*

§*Membrane Integrity, Danish Cancer Society Research Center, Strandboulevarden 49, 2100 Copenhagen, Denmark and Department of Cellular and Molecular Medicine, Faculty of Health Sciences, University of Copenhagen, Blegdamsvej 3C, DK-2200 Copenhagen, Denmark*

E-mail: doostmohammadi@nbi.ku.dk; bendix@nbi.ku.dk

1 Additional Figures

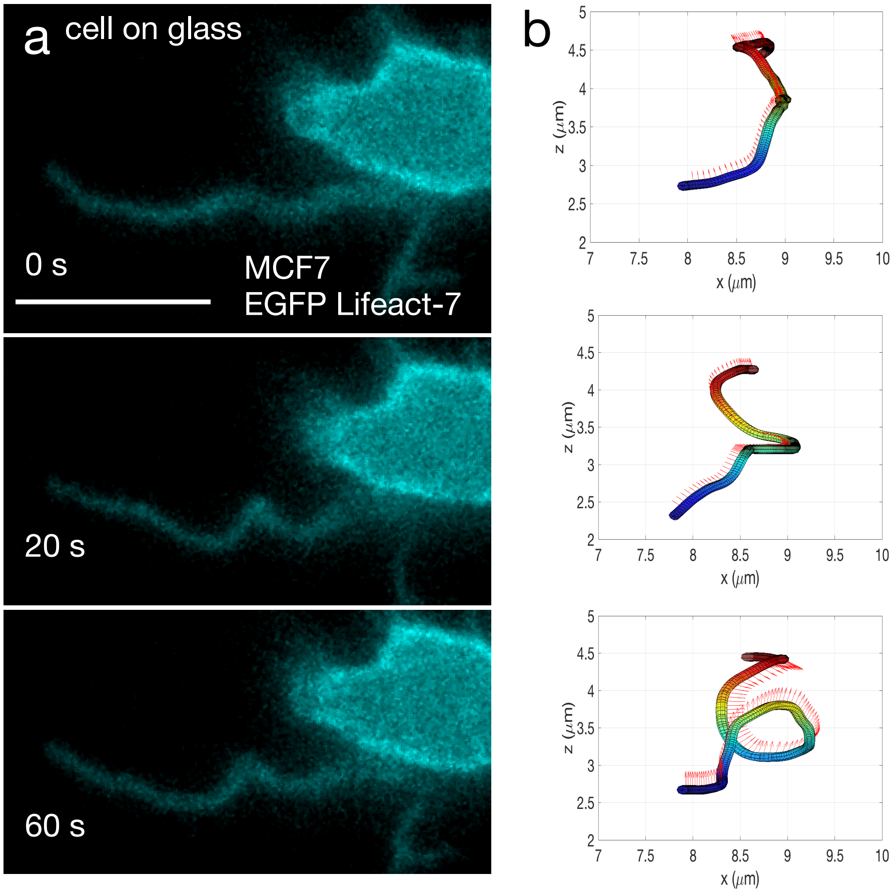

Supplementary Fig. 1 Further example of a buckling filopodium of a cell grown on glass. (a) Filopodium from a MCF7 cell (cyan, EGFP Lifeact-7) grown on glass at 3 different time points (at 0 s, 20 s and 60 s). Scale bar is 5 μm. (b) 3D tracks of the filopodium corresponding to the time points from (a) show how it bends and helically coils.

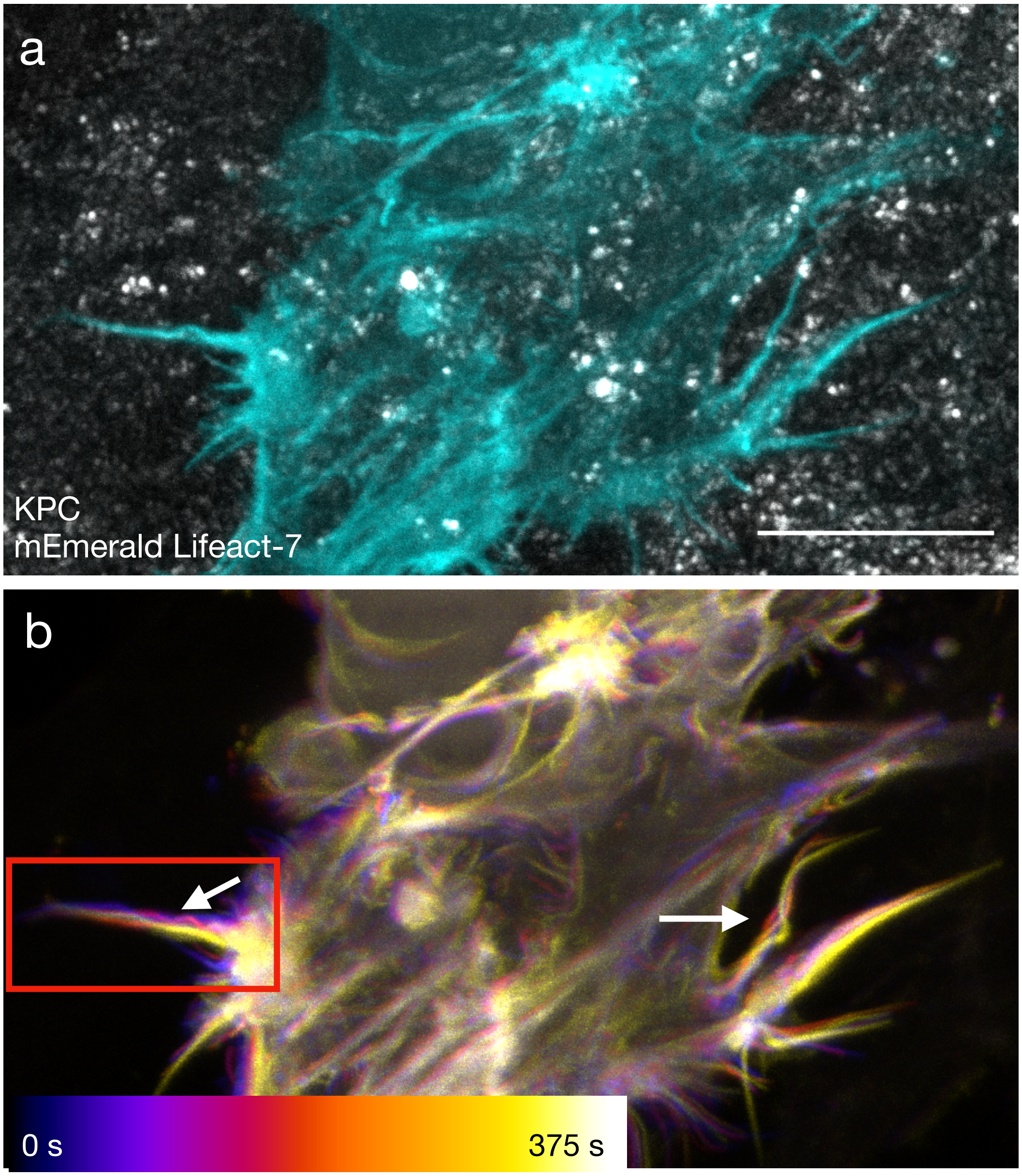

Supplementary Fig. 2. Further example of buckling filopodia from the KPC cell embedded in a 4 mg/ml collagen I gel from Fig. 1c,d. (a) Confocal Z-projection of a KPC cell (cyan, mEmerald Lifeact-7) in 4 mg/ml collagen I gel (gray, reflection). Scale bar is 10 μm. (b) Color coded temporal development of the cell from (a and Fig. 1c,d). Overlay of 6 Z-projections acquired at different time points over an interval of 375 s. Time resolution of each Z-stack is 15 s. The arrows highlight buckles. The right arrow marks the region shown in Fig.1 c,d. Brightness/contrast of the color channels (reflection and fluorescence)

were adjusted individually.

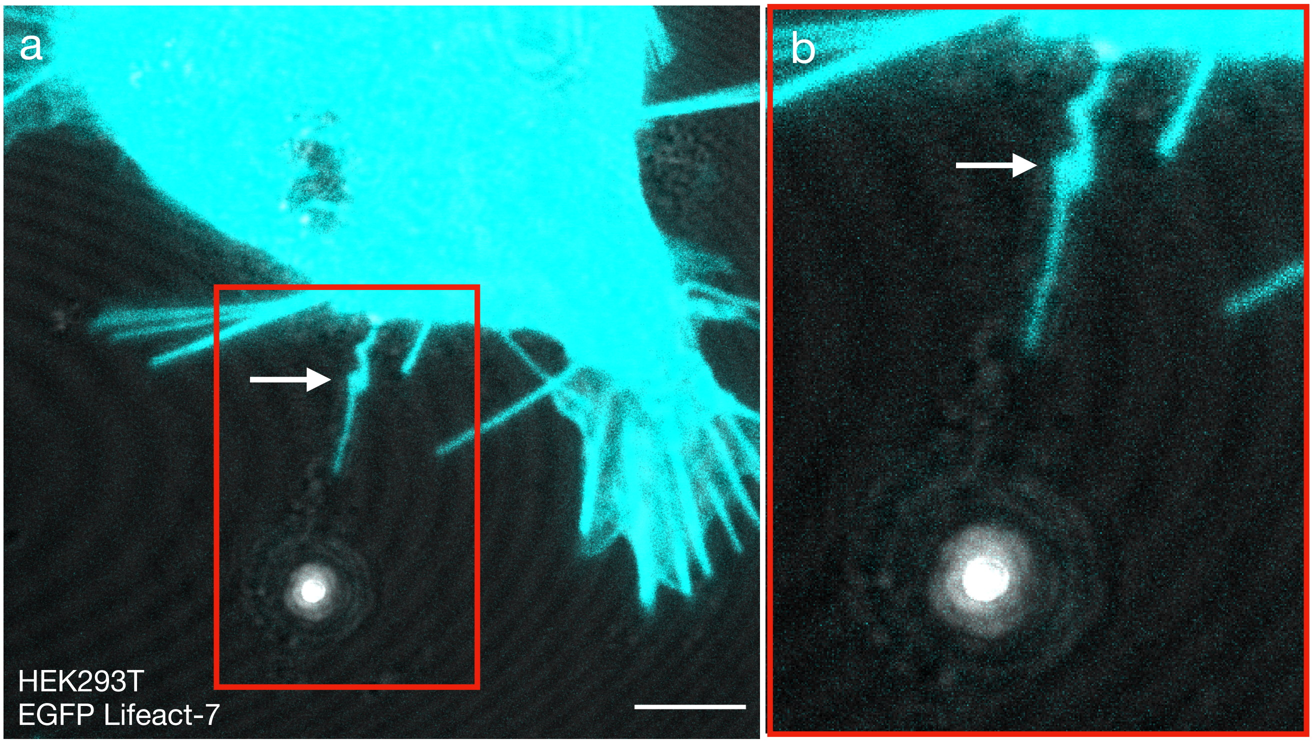

Supplementary Fig. 3 Filopodia rotation is not an artifact of confocal laser illumination. (Data presented in this Figure is the same as in Fig. 7j.) (a) Confocal Z-projection of tether extracted from a HEK293T cell (cyan, EGFP Lifeact-7) using a 4.96 μm bead shows a buckle (white arrow) stemming from rotation of the filopodium. The tether was extracted in bright field mode without exposing the cell to laser illumination prior to confocal imaging. Scale bar is 5 μm. (b) Zoom in to the red marked region in (a). Brightness/contrast of the color channels (reflection and fluorescence) were adjusted individually.

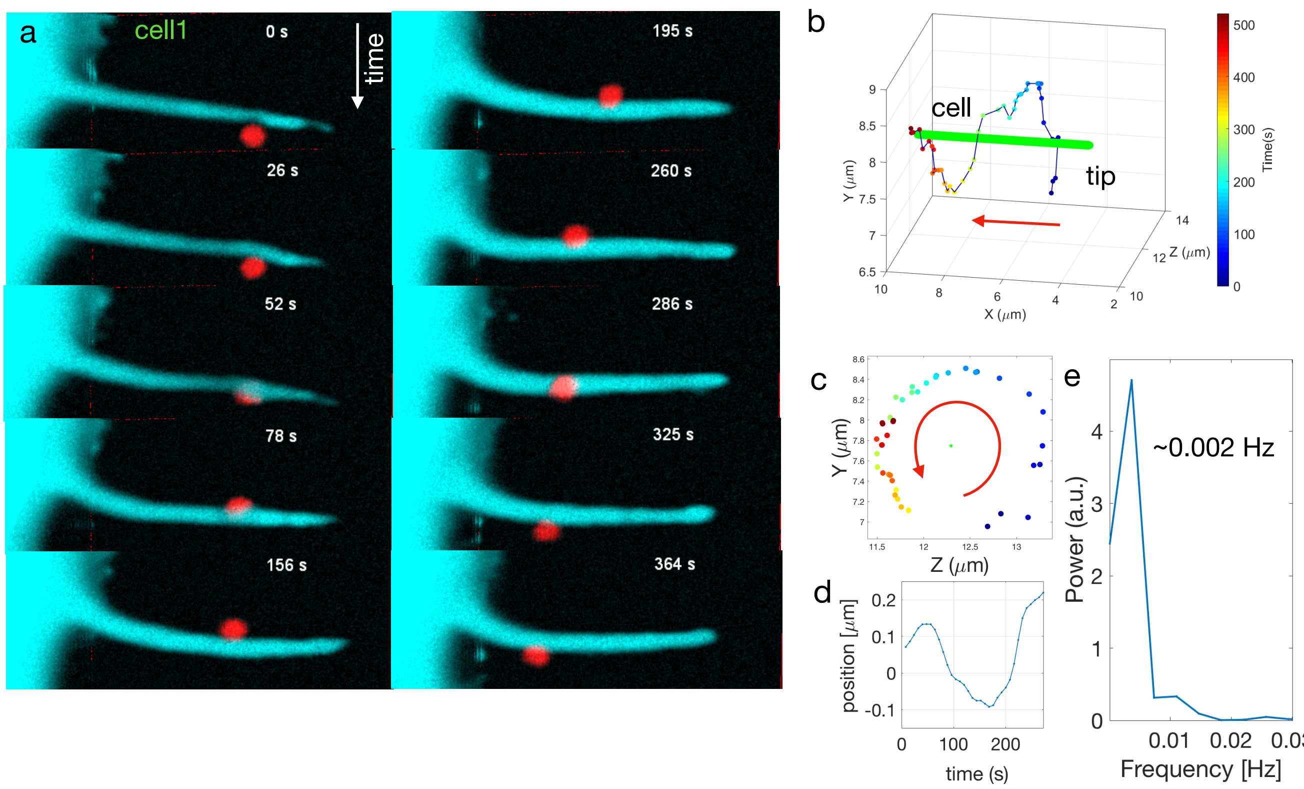

Supplementary Fig. 4 Bead rotation assay reveals that the F-actin shaft inside filopodia rotates around its own axis (additional data for cell 1 from Fig. 2b). (a) 3D reconstructed images of the rotation of a VN coated tracer bead ($d = 0.99$ μm, flash red) around an extended filopodium of a HEK293T cell (cyan, EGFP Lifeact-7, cell 1 from Fig. 2b) at consecutive time points. (b) 3D trajectory of the bead in (a) (from blue to red) rotating in an counterclockwise orientation around the filopodium (green) from tip towards the cell body. (c) XZ view of the bead movement from (a) over time, time bar is the same as in (b) shows the counterclockwise rotation (seen from tip towards the cell body). (d,e) The bead position (data from a) as a function of time (d) can be Fourier transformed to give the rotation frequency of the tracer bead 0.002 Hz.

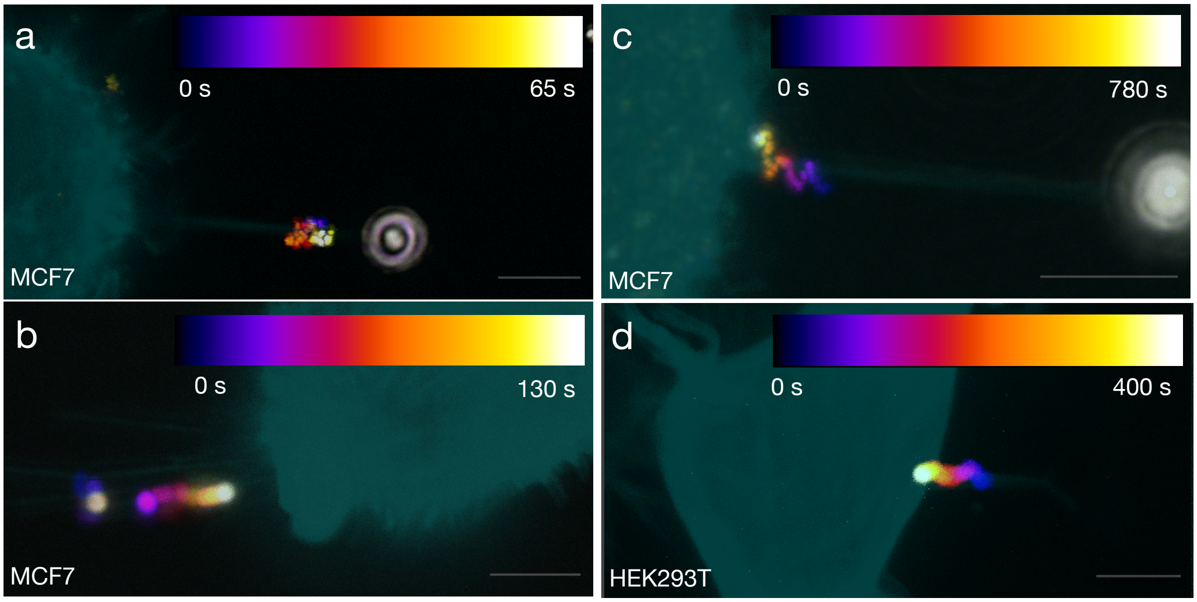

Supplementary Fig. 5 Further examples of a bead rotating around or moving along a filopodium. (a-d) Color coded Z-projection over time of single beads rotating around (a-c) MCF7 cells (cyan, EGFP Lifeact-7) and (d) a HEK293T cell (cyan, EGFP Lifeact-7). Scale bars are 5 μm.

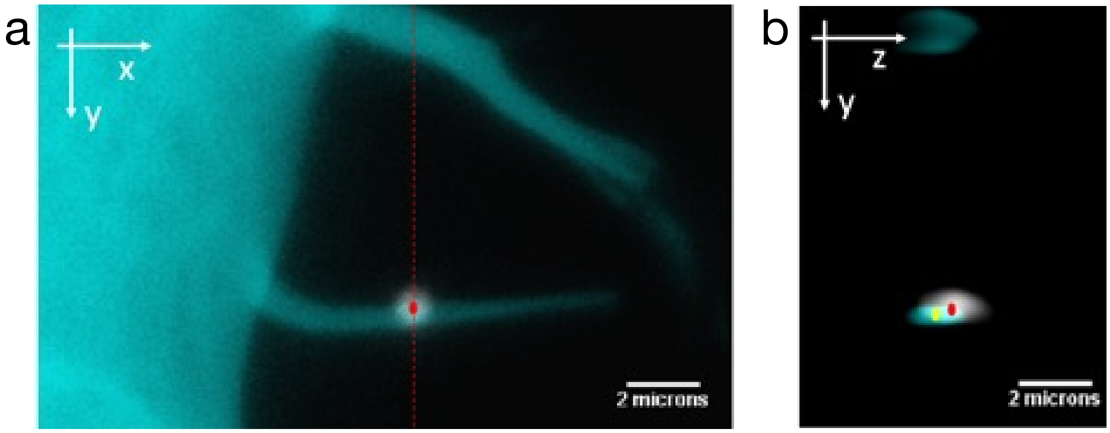

Supplementary Fig. 6 Tracking the bead movement along a filopodium. (a) The XY-position of a bead on a filopodium was extracted from the Z-projection image (red point). (b) The orthogonal view along red dashed line in a was used to localize the YZ-positions of the bead (red point) and center of the filopodium (yellow point).

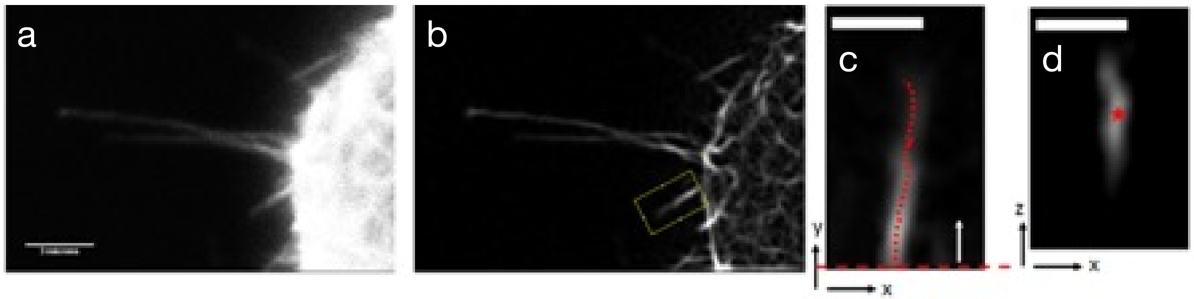

Supplementary Fig. 7 Filopodial tip tracking. (a) Z-projection of 3-dimensional volume of raw confocal images of a cell with filopodia. (b) The same image from a after applying linear Gabor filter to enhance the resolution. (c) The XY position of the desired filopodium (yellow box in b) was found by scanning the red dashed line along filopodium and applying a Gaussian fit to the intensity profile. (d) The Z location of the filopodium in each point (red points in c) was extracted from the orthogonal view of the image.

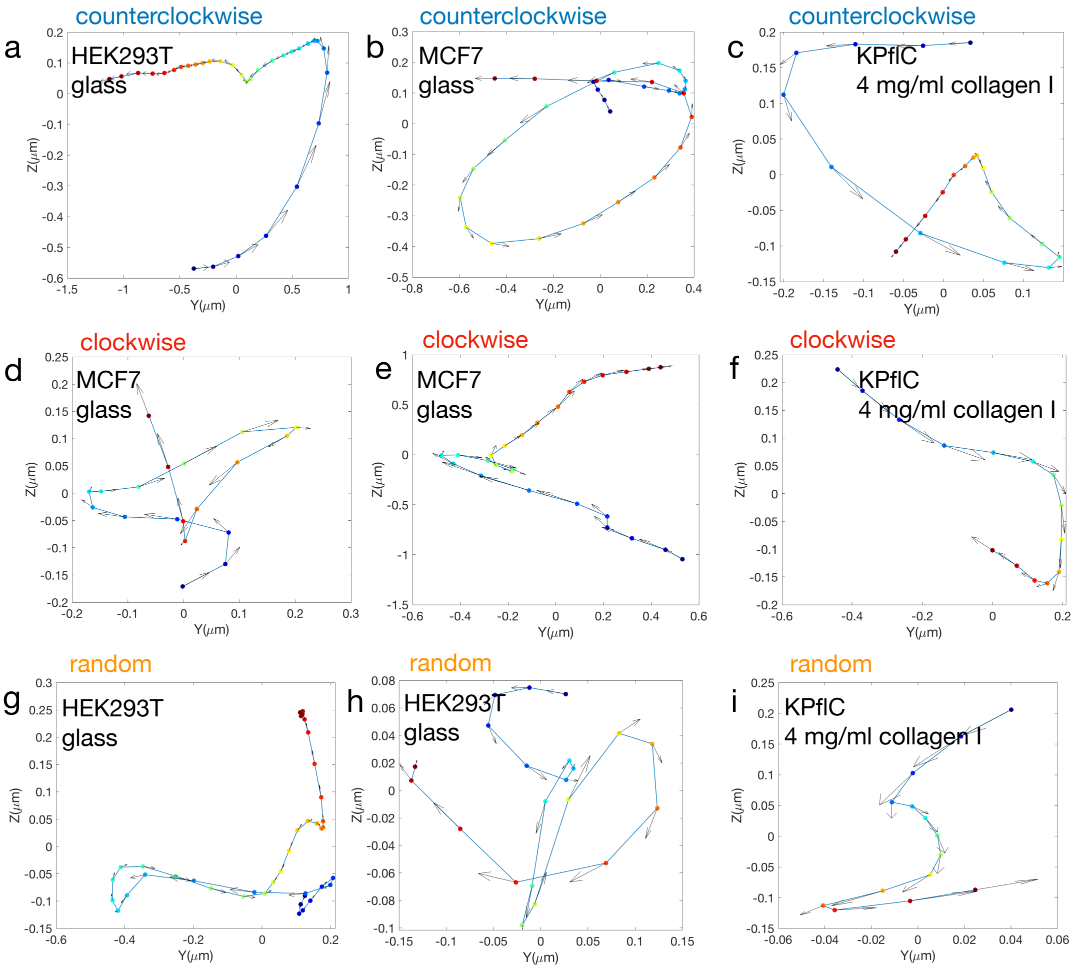

Supplementary Fig. 8 Further examples of filopodial tip rotation tracks. 51 % of tracked rotations were counterclockwise, 9 % clockwise, and 40 % random ($N = 68$). (a-c) Traces of the filopodial tip movement over time showing a counterclockwise rotation in YZ view for the tip of a HEK293T cell on glass (a), a MCF7 cell on glass (b), and a KP^{fl}C cell in 4 mg/ml collagen I (c). (d-f) Traces of the filopodial tip over time showing a clockwise rotation in YZ view for the tip of MCF7 cells on glass (d,e), and a KP^{fl}C cell in 4 mg/ml collagen I (f). (g,h,i) Traces of the filopodial tip over time showing random rotations in YZ view for the tip of HEK293T cells on glass (g,h), and a KP^{fl}C cell in 4 mg/ml collagen I (i).

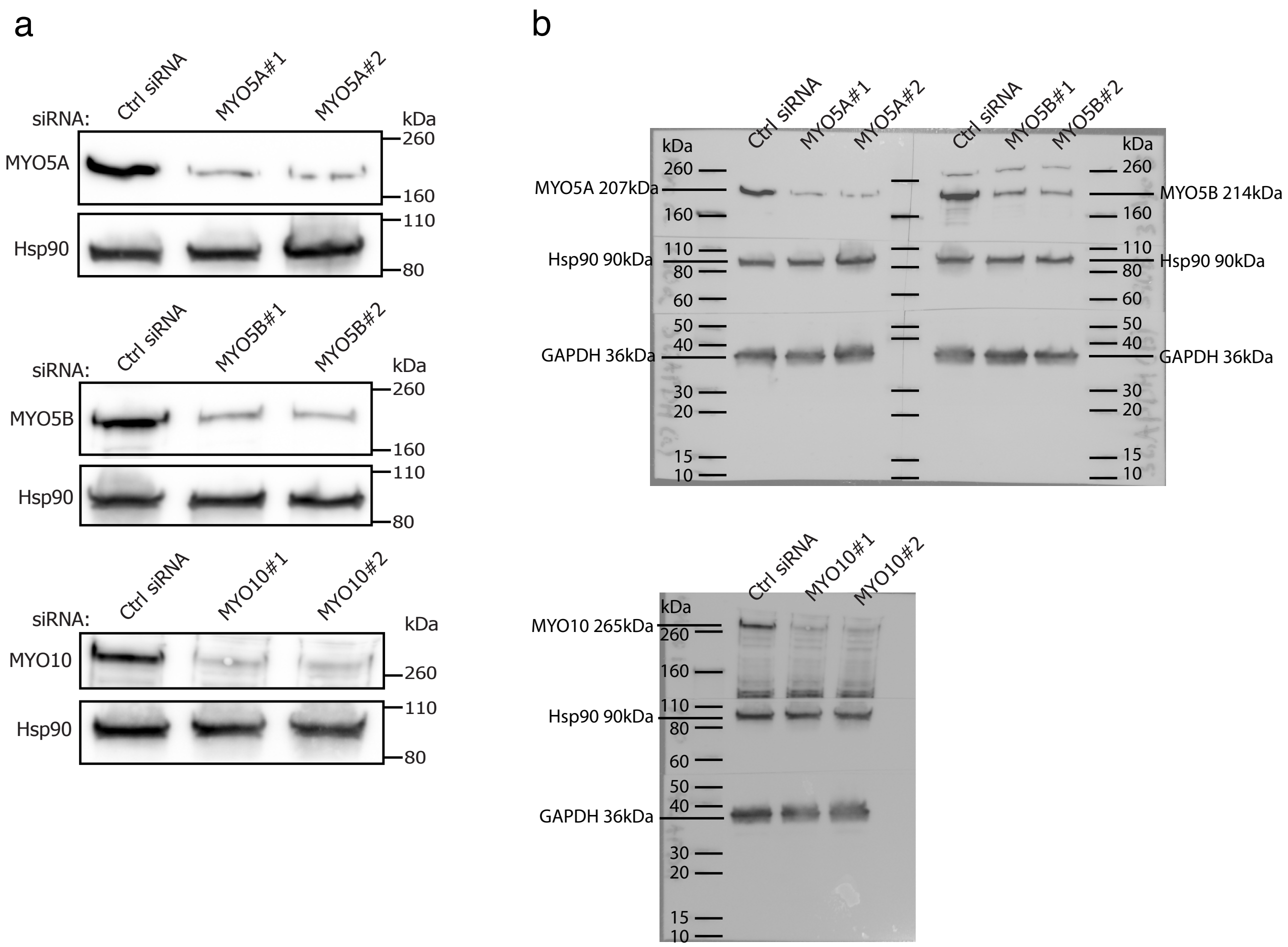

Supplementary Fig. 9 (a) Immunoblot of lysates from MCF7 cells 72 h after siRNA transfection with indicated siRNAs (MYO5A: 207 kDa, MYO5B 214 kDa, MYO10 265 kDa, Hsp90: loading control). (b) Extended blots. Experiments were repeated 3 times.

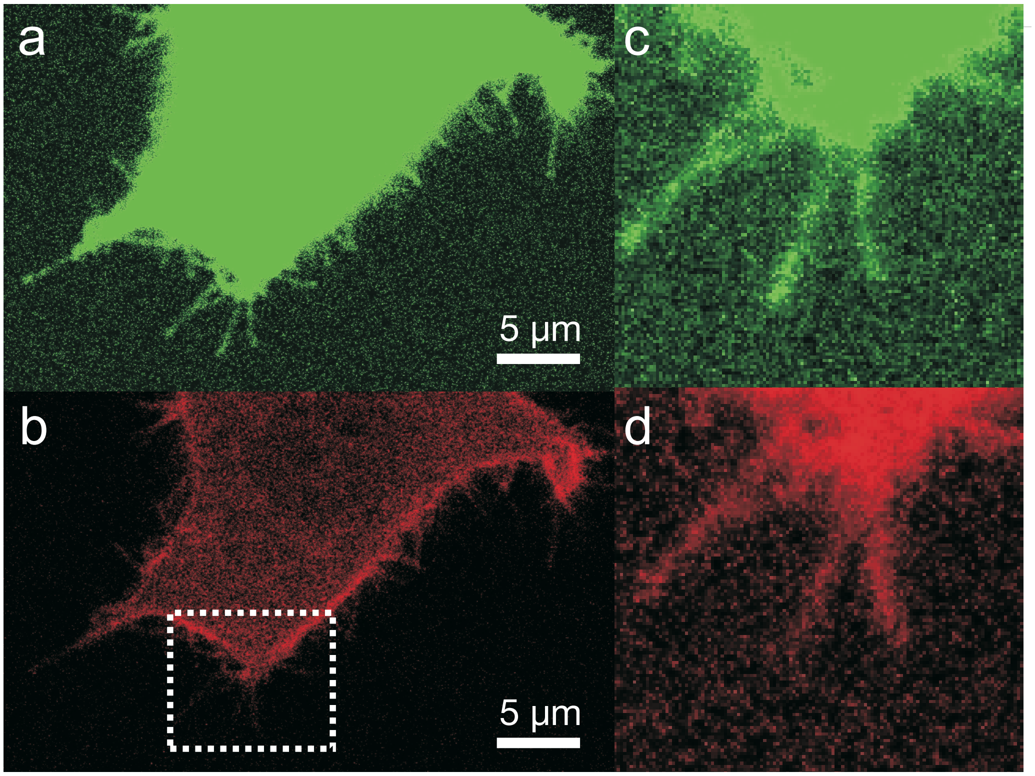

Supplementary Fig. 10 Confocal image of a MCF7 cell expressing (a) mEmerald-mDia1 (green) and (b) Lifeact-mCherry (red). (c,d) Enlarged view of the boxed region shown in (b). Representative data from 5 cells within 1 sample.

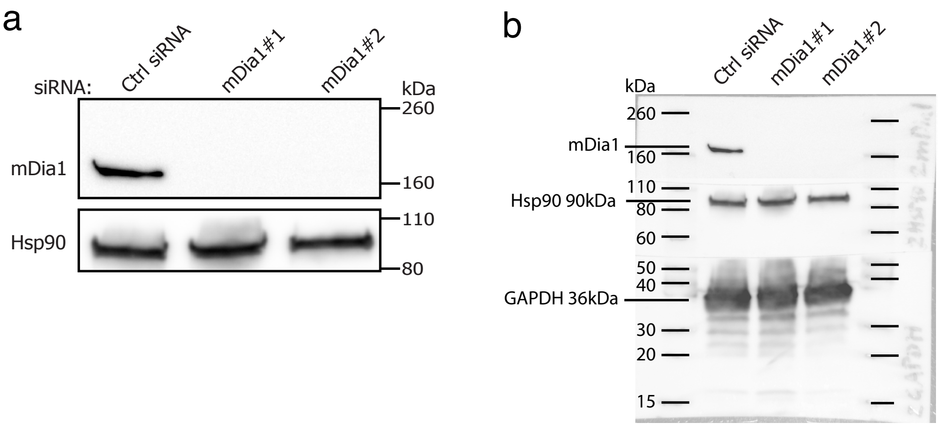

Supplementary Fig. 11 (a) Immunoblot of lysates from MCF7 cells 72 h after siRNA transfection with indicated siRNAs (mDia1: 170 kDa, Hsp90: loading control). (b) Extended blot. Experiments were repeated 3 times.

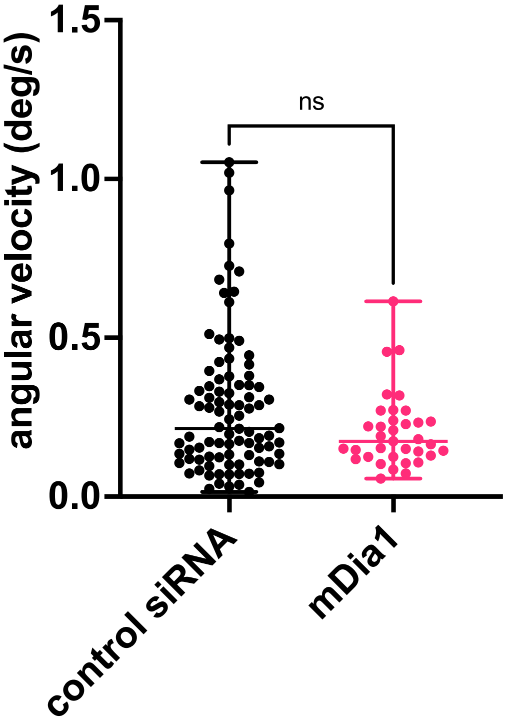

Supplementary Fig. 12 Results from silencing mDia1 by siRNA mDia1#1 in MCF7 cells on the angular velocities of individual filopodia compared with MCF7 cells transfected with control siRNA. There is no significant difference between the two populations (p-value = 0.1773, two tailed Mann-Whitney test). Scatter plot shows the median and the whiskers extend from minimum to the maximum values. N (filopodia) = 92 (control siRNA), 35 (mDia1).

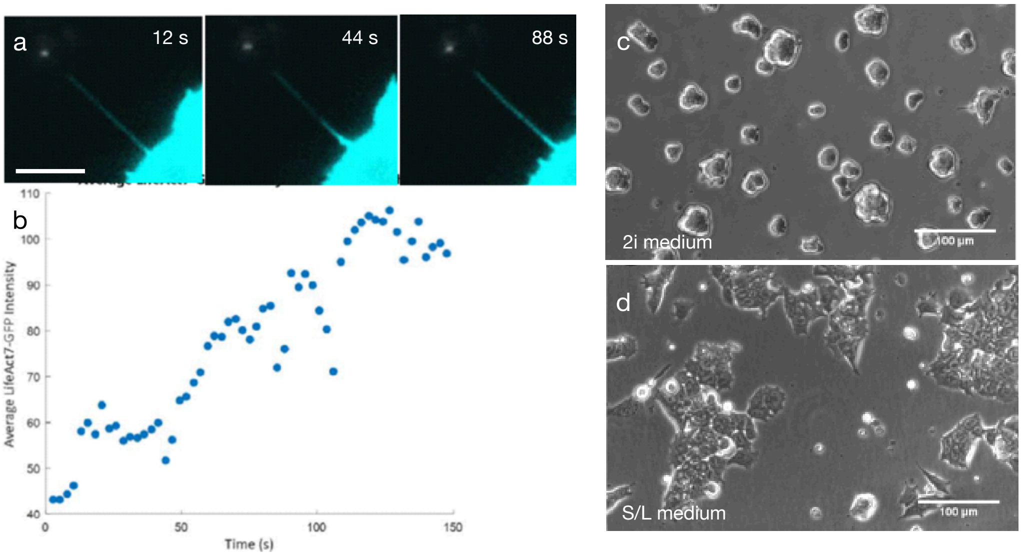

Supplementary Fig. 13 Tethers from stem cells show rich actin dynamics. (a) Tether extracted from a mES cell (cyan, EGFP Lifeact-7) grown in S/L medium at 3 different time points (12 s, 44 s, 88 s) after tether extraction. Scale bar is 5 μm. (b) Quantification of the F-actin content of the tether from a (via the average intensity of the EGFP Lifeact-7 signal) over a time of 180 s shows how F-actin accumulates inside the tether. (c and d) mES cells cultured in 2i medium (c) are less spread out than cells grown in S/L medium (d). Images are representative from 5 independent samples. Scale bars are 100 μm.

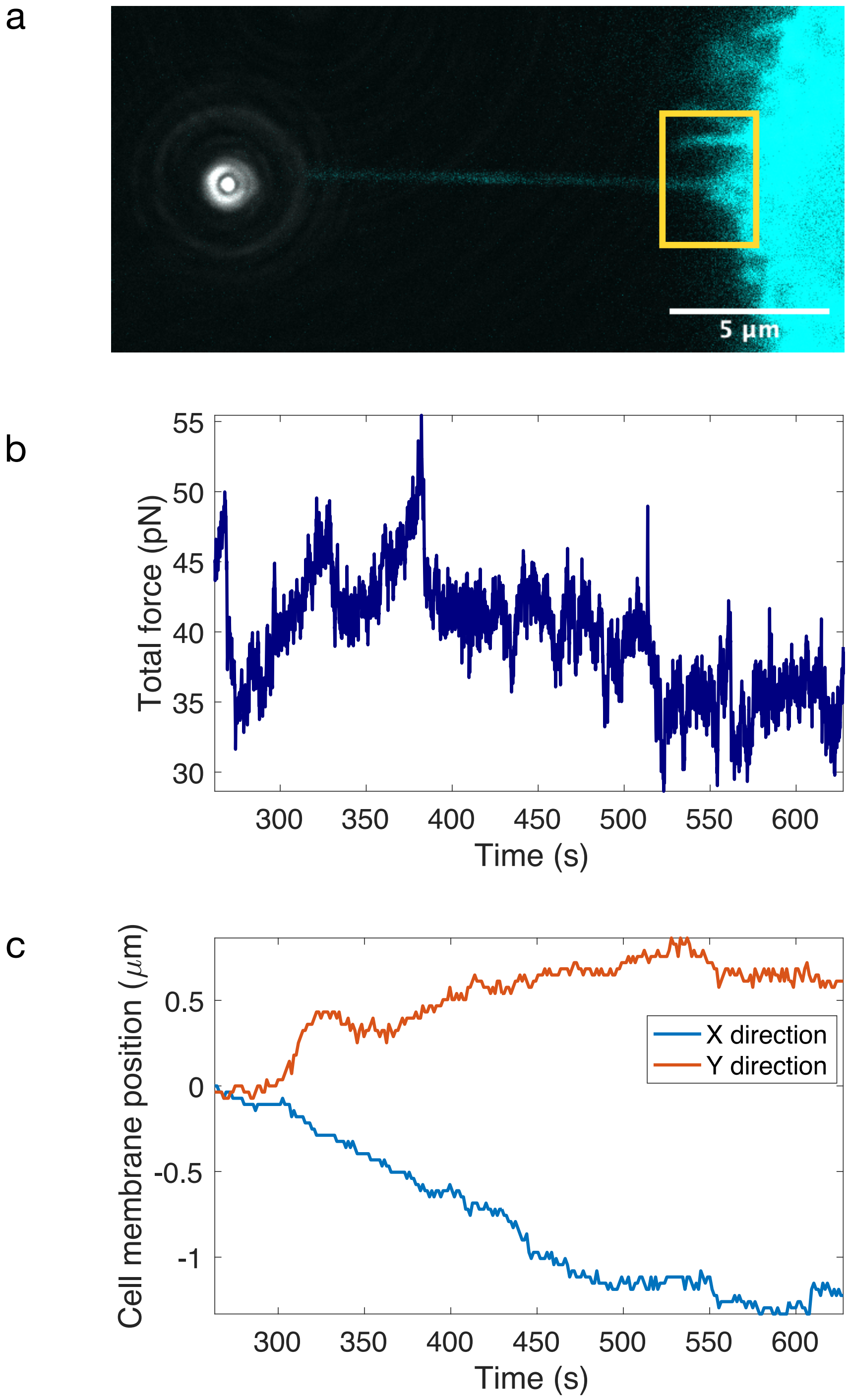

Supplementary Fig. 14 Tether force and cell movement. (a) Confocal image of a trapped bead (gray, reflection) holding a tether extracted from a cell (cyan, F-actin). Scale bar is 5 μm. The yellow rectangle marks the region for cell movement tracking described in (c).

(b) Total holding force required to hold the bead attached to the tether shown in (a). Time in (b) and (c) denotes the time after onset of the experiment where the tether was extracted (extraction data not shown). (c) Quantification of the cell body movement (tracked inside the yellow rectangle in a during the experiment in X and Y direction. Quantification of cell movement has been measured 2 times. Brightness/contrast of the color channels (reflection and fluorescence) were adjusted individually.

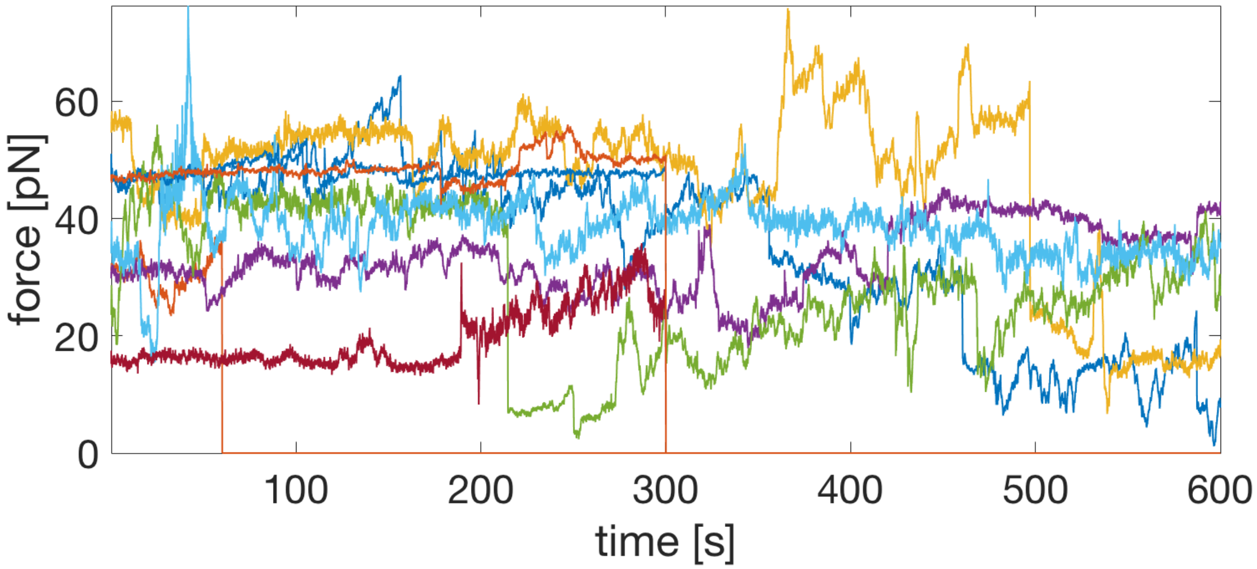

Supplementary Fig. 15 Tethers extracted from MCF7 cells show rich dynamics. 9 different force curves of tethers from MCF7 cells extracted with and held by an optically trapped bead ($d = 4.95\ \mu$m). Acquisition of the forces started after tether extraction.

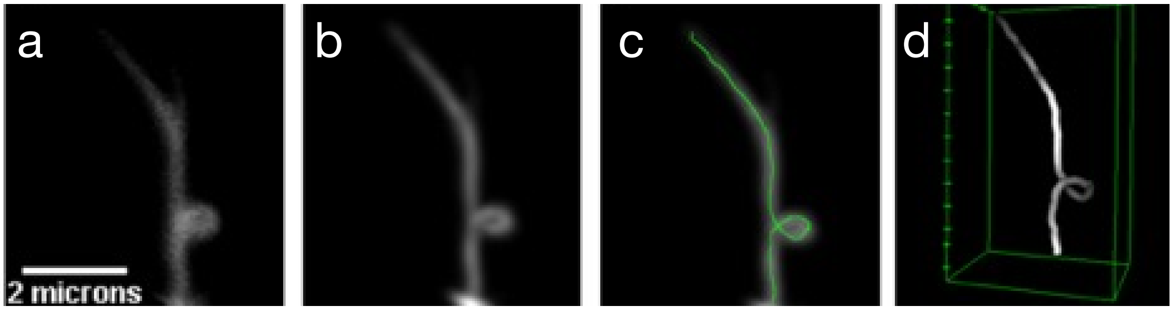

Supplementary Fig. 16 Tracking helical coils. (a) Z-projection of the 3-dimensional volume containing a coiling filopodium. (b) Gabor and Gaussian blur filtered result of the image from a. (c and d) Segmentation of the filopodium in 2D (c) and in a 3D (d) view; both obtained using the “Simple Neurite Tracer” plugin in ImageJ.

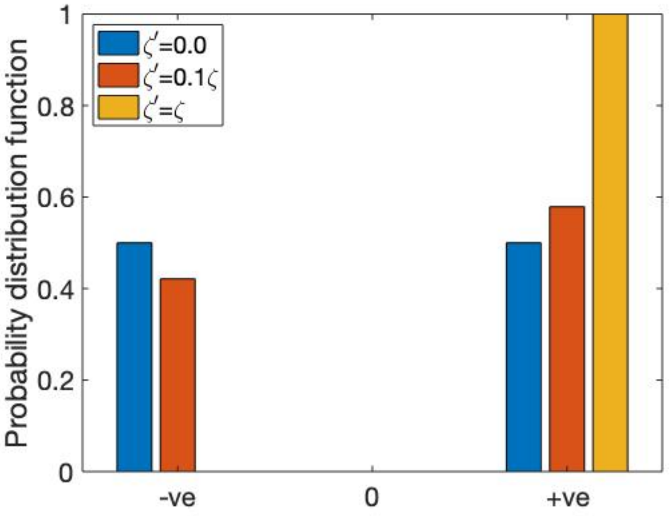

Supplementary Fig. 17 Probability distribution functions for the direction of rotation. Increasing the magnitude of the active torque dipole ζ' increases the biased rotation. Positive (negative) values are indicated by $+ve$ ($-ve$).

Supplementary Tables

cell type	bead rotation frequency (Hz)	bead velocity (μm/s)
**HEK293T**	0.002	0.0184
**HEK293T**	0.005	0.1222
**MCF7**	0.08	0.3676
**MCF7**	0.05	0.2309
**MCF7**	0.005	0.0312
**uninduced MCF7-p95ErbB2**	0.02	0.1297

Supplementary Table 1. Rotation frequencies and translational velocities for single tracer beads on filopodia of different cell types.

**cell type**	**N**	**mean angular velocity ± std. (deg/s)**
**MCF7 on glass**	14	0.53 ± 0.53
**HEK293T on glass**	22	0.30 ± 0.16
**MCF7-p95ErbB2 on glass**	4	0.23 ± 0.18
**KP^{fl}C in collagen**	23	0.11 ± 0.08
**KPC in collagen**	5	0.12 ± 0.09
**cells on glass**	40	0.37 ± 0.35
**cells in collagen**	28	0.11 ± 0.08

**cell types**	***p*-value**
**MCF7 (glass), MCF7-p95ErbB2 (glass)**	*** 0.0003
**HEK293T (glass), KP^{fl}C (collagen)**	*** 0.0005
**cells on glass, cells in collagen**	**** < 0.0001

Supplementary Table 2. Mean angular velocities for cells on glass and embedded in collagen I gels for the data in Figure 3i. 51 % of N = 68 filopodia (on glass and in collagen) showed counterclockwise, 9 % clockwise, and 40 % random rotation direction as seen from tip towards the cell body. The *p*-values for the not mentioned couples show no significant difference between the compared populations.

**Experiment 1**	N_{Filo}	CW (%)	CCW (%)	Random (%)	(mean ang. vel. ± std) (deg/s)
Control siRNA	31	23	45	32	0.29 ± 0.23
mDia1 siRNA	35	20	54	26	0.21 ± 0.12
MyoVa siRNA	23	22	26	52	0.11 ± 0.07
MyoVb siRNA	28	4	36	61	0.13 ± 0.15
MyoX siRNA	18	6	50	44	0.16 ± 0.14
**Experiment 2**					
Control siRNA	61	11	51	38	0.28 ± 0.22
MyoVa siRNA	65	14	18	68	0.14 ± 0.17

**cell type**	***p*-value mean angular velocity**
**control siRNA, MyoVa siRNA**	**** < 0.0001
**control siRNA, MyoVb siRNA**	*** 0.0003
**control siRNA, MyoX siRNA**	* 0.0171

Supplementary Table 3 Orientation of filopodia rotations before and after silencing of myosins and mDia1 by siRNAs (CW (clockwise), CCW (counterclockwise)). Both experiments are compared with cells transfected with control siRNA. MCF7 cells were transfected with Lifeact GFP to visualize the filopodia. Angular rotation velocities of filopodia after silencing myosins Va, Vb or myosin X, by siRNAs using myoVa#1, myoVb#1 or myoX#1, respectively, were compared to cells transfected with control siRNA. Kruskal-Wallis test was used with significance set at $p < 0.05$.
