# Supplementary material for: Filopodia rotate and coil by actively generating twist in their actin shaft
Source: Nat Commun. 2022 Mar 28;13:1636. doi: 10.1038/s41467-022-28961-x (PMC8960877; doi:10.1038/s41467-022-28961-x)
Supplement: Supplementary file 3 — Description of additional Supplementary File [file 41467_2022_28961_MOESM3_ESM.pdf]

Supplementary Movie 1
Sweeping filopodia from the HEK293T cell (cyan, EGFP Lifeact-7) from Figure 1a,b over a time span of 400 s. Scale bar is 5 μm.

Supplementary Movie 2

Bending filopodia from the KPC cell (cyan, EGFP Lifeact-7) from Figure 1c,d and Supplementary Figure 2 in 4 mg/ml collagen I (gray, reflection) over a time span of 450 s. Red arrows highlight bending regions. Scale bar is 5 μm.

Supplementary Movie 3
3D reconstruction of confocal XYZt data showing how a bead travels along a filopodium. VN coated 0.99 μm bead (purple) rotating counterclockwise (as seen from tip towards cell body) around a pulled filopodium from a not activated MCF7-p95ErbB2 cell (cyan, EGFP Lifeact-7). The filopodium is held by a 4.95 μm VN coated bead (gray, reflection) which is immobilized on the sample surface. Frame interval is 8sec, total time is 384 s.

Supplementary Movie 4 (same as Movie 3 without imaging of the surface-attached bead)
VN coated 0.99 μm bead (red) attached to the filopodial membrane of a MCF7 cell not linked to actin (cyan, EGFP Lifeact-7). The filopodium is held by a 4.95 μm VN coated bead (not seen) which is glued to the sample surface. Frame interval is 0.39 s, total time is 114 s. Scale bar is 5 μm.

Supplementary Movie 5
Filopodia on a HV.5.1 mESC (cyan, EGFP Lifeact-7) grown in 2i medium.

Supplementary Movie 6
Rotating filopodia on Hepa 1-6 cell (red, SiR-actin) over 71.5 min, highlighted via a white arrow. Scale bar is 10 μm.

Supplementary Movie 7
Rotating filopodia on Hepa 1-6 cell (red, SiR-actin) over 58 min, highlighted via a white arrow. Scale bar is 10 μm.

Supplementary Movie 8
Bending filopodia from KPC cell (cyan, EGFP Lifeact-7) in 4 mg/ml collagen I (gray, reflection) over a time span of 399 s. Red arrows highlight bending regions. Scale bar is 10 μm.

Supplementary Movie 9
Coiling filopodium from the MCF7 cell (cyan, EGFP Lifeact-7) from Supplementary Figure 1, 7d,e over 160 s. Scale bar is 5 μm.

Supplementary Movie 10
Tether pulling from a MCF7 cell (cyan, EGFP Lifeact-7) using a VN coated 4.95 μm bead (grey, reflection). After extraction, the image zoomed in and the focus is adjusted such that the tether is aligned with the imaging plane. Total time is 450 s. Scale bar is 5 μm.

Supplementary Movie 11
Bending tether pulled from a HV.5.1 mESC (cyan, EGFP Lifeact-7) using a VN coated 4.95 μm bead (grey, reflection).
