# Supplementary material for: Filopodia rotate and coil by actively generating twist in their actin shaft
Source: Nat Commun. 2022 Mar 28;13:1636. doi: 10.1038/s41467-022-28961-x (PMC8960877; doi:10.1038/s41467-022-28961-x)
Supplement: Supplementary file 15 — Reporting Summary [file 41467_2022_28961_MOESM15_ESM.pdf]

nature research

Corresponding author(s): *Double-blind peer review submissions: write DBPR and your manuscript number here instead of author names.*

Last updated by author(s): *YYYY-MM-DD*

Reporting Summary

Nature Research wishes to improve the reproducibility of the work that we publish. This form provides structure for consistency and transparency in reporting. For further information on Nature Research policies, see our Editorial Policies and the Editorial Policy Checklist.

Statistics

For all statistical analyses, confirm that the following items are present in the figure legend, table legend, main text, or Methods section.

n/a	Confirmed	
☐	☒	The exact sample size (*n*) for each experimental group/condition, given as a discrete number and unit of measurement
☒	☐	A statement on whether measurements were taken from distinct samples or whether the same sample was measured repeatedly
☐	☒	The statistical test(s) used AND whether they are one- or two-sided<br>*Only common tests should be described solely by name; describe more complex techniques in the Methods section.*
☒	☐	A description of all covariates tested
☐	☒	A description of any assumptions or corrections, such as tests of normality and adjustment for multiple comparisons
☐	☒	A full description of the statistical parameters including central tendency (e.g. means) or other basic estimates (e.g. regression coefficient) AND variation (e.g. standard deviation) or associated estimates of uncertainty (e.g. confidence intervals)
☐	☒	For null hypothesis testing, the test statistic (e.g. *F*, *t*, *r*) with confidence intervals, effect sizes, degrees of freedom and *P* value noted<br>*Give P values as exact values whenever suitable.*
☒	☐	For Bayesian analysis, information on the choice of priors and Markov chain Monte Carlo settings
☒	☐	For hierarchical and complex designs, identification of the appropriate level for tests and full reporting of outcomes
☒	☐	Estimates of effect sizes (e.g. Cohen's *d*, Pearson's *r*), indicating how they were calculated

Our web collection on statistics for biologists contains articles on many of the points above.

Software and code

Policy information about availability of computer code

Data collection

Labview was used to collect data from the optical trap (photodiode). Voltage signals were acquired for the x,y,z position and stored as dat files to be analyzed by Matlab software (see next section). LAS leica software installed on a Leica SP5 TCS confocal system, was used to acquire confocal images and z-stacks and stored as *.LIF files. LIF files were then loaded into Fiji/Image J (free online software) and exported as TIFF stacks which were analyzed by Matlab.

Data analysis

Images of filopodia are acquired as confocal z-stacks at consequetive time points. These stacks are subsequently analyzed by Fiji and codes written in Matlab software (Matlab 2017a). The 3D tracks of filopodia were plotted as 3D figures in Matlab.
Forces from filopodia were acquired as position displacements of beads attached to the filopodium tip over time. The optical trap holding the bead has a spring like potential and hence the displacements were multiplied by the calibrated optical trap constant. Calibration was performed using published Matlab software (Hansen et al. Computer Physics Communications, 174, 6, 518-520 (2006)).
Statistical analysis, plots and p-values were were analyzed using the Graphpad Prism software (version 9.2.0) with a default 5% significance level. The Matlab codes used for analyzing the data in this work are available through github:\\[https://github.com/Younes-FB/Gabor_image_filtering] (Image filtering).[https://github.com/Younes-FB/filament_3Dtrack] (Code for 3D tracking of filopodia).[https://github.com/Younes-FB/Track_bead_On_tube_3D] (Code for tracking beads on filopodia).

For manuscripts utilizing custom algorithms or software that are central to the research but not yet described in published literature, software must be made available to editors and reviewers. We strongly encourage code deposition in a community repository (e.g. GitHub). See the Nature Research guidelines for submitting code & software for further information.

Data

Policy information about availability of data

All manuscripts must include a data availability statement. This statement should provide the following information, where applicable:

- Accession codes, unique identifiers, or web links for publicly available datasets
- A list of figures that have associated raw data
- A description of any restrictions on data availability

Data for all figures are provided in the Data Source File. Representative raw data sets are provided together with the code at Github (see above). The whole raw pool of data is too extenstive to upload with sufficient instructions. There we recommend researchers to contact the corresponding authors for raw data and information on the data. The data that support the findings of this study are available from the corresponding author (experiments: bendix@nbi.dk, and simulations:doostmohammadi@nbi.ku.dk) upon reasonable request

Field-specific reporting

Please select the one below that is the best fit for your research. If you are not sure, read the appropriate sections before making your selection.

☒ Life sciences ☐ Behavioural & social sciences ☐ Ecological, evolutionary & environmental sciences

For a reference copy of the document with all sections, see nature.com/documents/nr-reporting-summary-flat.pdf

Life sciences study design

All studies must disclose on these points even when the disclosure is negative.

Sample size	We have stated the how many filopodia we have measured on in the figure legends or in the statistical analysis section.
Data exclusions	Only cells which can be accurately quantified are included in the analysis. Some cells drifted out of focus and occasionally signals were too weak to perform long time imaging - such cases are therefore not included.
Replication	Replication of experiments depends on the type of experiment conducted. The knock-down study in fig. 3 was carried out twice where as the other experiments were replicated a number of times (>3)
Randomization	Experiments were conducted on different cell lines on random days.
Blinding	Blinding is not relevant for this type of experiments. Each researcher needs to control all aspects of a single experiment possibly in collaboration with another researcher.

Reporting for specific materials, systems and methods

We require information from authors about some types of materials, experimental systems and methods used in many studies. Here, indicate whether each material, system or method listed is relevant to your study. If you are not sure if a list item applies to your research, read the appropriate section before selecting a response.

Materials & experimental systems

n/a	Involved in the study	
☐	☒	Antibodies
☐	☒	Eukaryotic cell lines
☒	☐	Palaeontology and archaeology
☒	☐	Animals and other organisms
☒	☐	Human research participants
☒	☐	Clinical data
☒	☐	Dual use research of concern

Methods

n/a	Involved in the study	
☒	☐	ChIP-seq
☒	☐	Flow cytometry
☒	☐	MRI-based neuroimaging

Antibodies

Antibodies used	mDia1: Purified Mouse Anti-mDia1, clone51/mDia1(RUO) BD Biosciences #610848<br>Myosin Va: MYO5A Rabbit, Cell Signaling Technology #3402<br>Myosin Vb: MYO5B, Rabbit, Novus Biologicals NBP1-87746<br>Myosin 10: MYO10, Rabbit, Sigma Aldrich HPA024223, LOT R11422<br>Hsp90: Purified Mouse Anti-Hsp90, Clone 68/Hsp90, BD Biosciences #610418, Lot 7341586<br>GAPDH: GAPDH Mouse mAb, clone 4G5, Abcam ab189095, Lot GR3260555-4<br>peroxidase-conjugated secondary antibodies:

Goat Anti-rabbit immunoglobulin G (IgG), Vector Laboratories #PI-1000

Rabbit Anti-mouse IgG, Dako #P0260

Validation

mDia1: BD Biosciences routinely tests the antibody for Western blot application. No knockdown or knockout controls are provided. In our experiments, the antibody recognized an antigen around 170 kDa in MCF7 cells and Ctrl siRNA-treated MCF7, while no signal was detected in mDia1 siRNA-treated MCF7 cells (see Supplementary Fig. 11). References provided by BD Biosciences:
(1) Li F, Higgs HN. The mouse Formin mDia1 is a potent actin nucleation factor regulated by autoinhibition. Curr Biol. 2003; 13(15):1335-1340.(Biology)
(2) Pennisi E. The architecture of hearing. Science. 1997; 278(5341):1223-1224.(Biology)
(3) Watanabe N, Madaule P, Reid T. p140mDia, a mammalian homolog of Drosophila diaphanous, is a target protein for Rho small GTPase and is a ligand for profilin. EMBO J. 1997; 16(11):3044-3056.(Biology)

Myosin Va: Cell signaling provides data on Western blotting applications: Myosin Va antibody detects endogenous levels of total myosin Va heavy chain (207 kDa). Based on sequence homology, the antibody is expected to detect all known myosin Va splice variants. No knockdown or knockout controls are provided. In our experiments, the antibody recognized an antigen around 207 kDa in MCF7 cells and Ctrl siRNA-treated MCF7, while less signal was detected in MYO5A siRNA-treated MCF7 cells (see Supplementary Fig. 9). 17 articles cite this antibody (For details see https://www.citeab.com/antibodies/122847-3402-myosin-va-antibody?des=f8db05e34a1f38d1).
(1) Zhi, Z., et al. (2021) Cell Death Differ
(2) Zhang, J., et al. (2020) Nature
(3) Jo, C. S., et al. (2020) Theranostics, 10, 3880-3891

Myosin Vb: Novus Biologicals provides data on Western blotting applications. No knockdown or knockout controls are provided. In our experiments, the antibody recognized an antigen around 214 kDa in MCF7 cells and Ctrl siRNA-treated MCF7, while less signal was detected in MYO5A siRNA-treated MCF7 cells (see Supplementary Fig. 9). References provided by Novus Biologicals:
(1) Royo, M., et al. (2019) J Cell Sci [PMID: 31757887]
(2) Engevik A. C., et al. (2020) Gastroenterology [PMID: 32112796]
(3) Forteza R., et al. (2019) Mol. Biol. Cell [PMID: 31664880]
7 articles in total site this antibody (for more details see https://www.citeab.com/antibodies/490124-nbp1-87746-myosin-vb-antibody?des=9d58310086077ec5).

Myosin 10: Sigma Aldrich has tested the antibody in Western blotting. Anti-MYO10 antibody produced in rabbit Prestige Antibodies® Powered by Atlas Antibodies. No knockdown or knockout controls are provided. In our experiments, the antibody recognized an antigen around 265 kDa in MCF7 cells and Ctrl siRNA-treated MCF7, while less signal was detected in MYO10 siRNA-treated MCF7 cells (see Supplementary Fig. 9). References provided by Sigma Aldrich:
(1) Chen C.P., et al. (2016) Oncology Reports, 35, 709-716
(2) Wan-Hsin L., et al. (2013) Journal of cell science, 126(Pt 20), 4756-4768
(3) Lin L., et al. (2013) Nature communications, 4, 2270-2270
28 articles in total site this antibody (for more details see https://www.sigmaaldrich.com/DK/en/search/hpa024223?focus=papers&page=1&perPage=30&sort=relevance&term=HPA024223&type=citation_search and https://www.citeab.com/antibodies/1516208-hpa024223-anti-myo10-antibody-produced-in-rabbit?des=4cd3e490b1827f9c).

Hsp90: BD Bioscience has tested the antibody reactivity towards a 90 kDa antigen in Western blotting. No knockdown or knockout controls are provided. In our experiments, the antibody was used as a loading control and detects a 90 kDa antigen (see Supplementary Fig. 9, 11). References provided by BD Biosciences:
Brouet A., et al. (2001), Hsp90 ensures the transition from the early Ca2+-dependent to the late phosphorylation-dependent activation of the endothelial nitric-oxide synthase in vascular endothelial growth factor-exposed endothelial cells. J Biol Chem., 276(35):32663-32669.
Miyamoto A., et al. (2002), Increased proliferation of B cells and auto-immunity in mice lacking protein kinase Cdelta, Nature, 416(6883):865-869.
Murata T., et al. (2002), Decreased endothelial nitric-oxide synthase (eNOS) activity resulting from abnormal interaction between eNOS and its regulatory proteins in hypoxia-induced pulmonary hypertension, J Biol Chem., 277(46):44085-44092.
Pritchard KA Jr and Ackerman AW. (2001), Heat shock protein 90 mediates the balance of nitric oxide and superoxide anion from endothelial nitric-oxide synthase, Chem., 276(21):17621-17624.

GAPDH: Abcam has tested the antibody reactivity towards a 36 kDa antigen in Western blotting. No knockdown or knockout controls are provided. In our experiments, the antibody was used as a loading control and detects a 36kDa antigen (see Supplementary Fig. 9 and 11). References provided by Abcam:
Crotti A., et al. (2019), BIN1 favors the spreading of Tau via extracellular vesicles. Sci Rep 9:9477.
Sønder, S.L. et al. (2019) Annexin A7 is required for ESCRT III-mediated plasma membrane repair. Sci Rep, 9, pp. 6726.

Eukaryotic cell lines

Policy information about cell lines

Cell line source(s)

HEK293 (ATCC #CRL-1573) and HEK293T (ATCC #CRL-11268) were purchased at ATCC, Stem cell line HV.5.1 was obtained from the Brickman Group (DanStem, University of Copenhagen, Denmark), KPR172HC and KPflC pancreatic cancer cell lines were a kind gift from Jennifer Morton (Beatson Institute), MCF7 and MCF7-p95ErbB2 were obtained from Jesper Nylandsted at the Danish Cancer Research Society Center.

MCF7 S1: originated from MCF7S cell line provided by Dr. David Springs, University of Wisconsin, cell line generation published in "Jaattela, M., Benedict, M., Tewari, M., Shayman, J.A. & Dixit, V.M. Bcl-x and Bcl-2 inhibit TNF and Fas-induced apoptosis and activation of phospholipase A2 in breast carcinoma cells. Oncogene 10, 2297-2305 (1995)".

MCF7-p95ErbB2 cell line is single cell clone of MCF7 cells stably expressing the tetracycline transactivator with a truncated version of ErbB2 (p95ErbB2) in the pTRE plasmid39. p95ErbB2 expression is induced by washing off tetracyclin (1µg/mL) with PBS. All cells were kept at 37°C in a humidified atmosphere of 5% CO_2. The experiments were carried out at 3–5 passage after the induction and in all the experiments with p95ErbB2 refers to induced cells.
More info in: Egeblad, M., Mortensen, O. H. & Jaattela, M. Truncated ErbB2 receptor enhances ErbB1 signaling and induces reversible, ERK-independent loss of epithelial morphology. Int.J.Cancer 94, 185–191 (2001).

Authentication

None of the cell lines used were authenticated

Mycoplasma contamination

Mycoplasma tests were regularly conducted by our collaborators with negative outcomes before we received the cells.

Commonly misidentified lines
(See ICLAC register)

None of the used cell lines are listed in the ICLAC register
