# Supplementary material for: Filopodia rotate and coil by actively generating twist in their actin shaft
Source: Nat Commun. 2022 Mar 28;13:1636. doi: 10.1038/s41467-022-28961-x (PMC8960877; doi:10.1038/s41467-022-28961-x)
Supplement: Supplementary file 16 — Source Data [file 41467_2022_28961_MOESM16_ESM.zip › How to access the data.docx]

# Explanatory file for opening the raw data files comprising the figures.

In general all data behind  **all figure-files (except for Figure 6, SI_Figure 9 and SI_Figure 5A,B** ) can be opened in any version of **Matlab (MathWorks).** Figures are in matlab format (.fig files) and by opening the figure property all data in the figure can be accessed.

We have also exported the data in the figures to .mat files which can be opened in Matlab and all numbers and variables can be readily accessed.

Figures are labeled with the respective number in the manuscript, including subpanels and insets.

**Figure 6** contains simulation data and data for Figure 6C,D,E are given as excel files.

**SI_Figure 5A,B** opens in Excel

**SI_Figure 9** opens in Excel
